# Supplementary material for: Are carbon emissions trading and green financial instruments synergistic? -Comprehensive quantitative research based on content analysis
Source: PLoS One. 2024 Mar 7;19(3):e0298601. doi: 10.1371/journal.pone.0298601 (PMC10919601; doi:10.1371/journal.pone.0298601)
Supplement: S1 Data — (ZIP) [file pone.0298601.s001.zip › Data/地级市Z_F工作报告.zip!/╣π╢1⁄2/├»├√╩╨/└┤╫╘╣1⁄2╓┌║┼í╛┬φ┐╦╩2╛▌═°í┐_2019.docx]

各位代表：

　　我代表市人民政府向大会作工作报告，请予审议，并请政协各位委员和其他列席人员提出意见。

　　一、2018年工作回顾

　　过去一年，是茂名以实干实绩迎接建市60周年的奋进之年，是聚焦新定位、担当新使命，奋力建设产业实力雄厚的现代化滨海城市、打造沿海经济带上的新增长极的开局之年。一年来，在省委、省政府和市委坚强领导下，在市人大、市政协监督支持下，市政府全面贯彻习近平新时代中国特色社会主义思想和党的十九大精神，深入落实习近平总书记对广东重要讲话和重要指示批示精神，坚持稳中求进工作总基调，统筹稳增长、促改革、调结构、惠民生、防风险，经济实现平稳健康发展，社会大局和谐稳定。

　　经济运行稳中有进。全年实现地区生产总值3092.18亿元，增长5.5%，突破3000亿元大关。来源于茂名的财政总收入560.57亿元，增长7.4%。地方一般公共预算收入136.14亿元，增长6.2%。社会消费品零售总额1541.37亿元，增长8.5%。规上工业经济效益综合指数550%，居全省首位。综合实力迈上新台阶，再度上榜“中国百强城市”。

　　产业发展加快突破。全年吸引国内外客商到茂名投资考察100多批次，新引进超千万元项目450个，深圳华侨城集团、新加坡鹏瑞利集团等行业领军企业落户茂名；丙烷脱氢产业链项目、益海嘉里粮油食品加工项目等优质产业项目布局滨海新区。

　　城市发展提质升级。深茂铁路如期通车，茂名进入“动车时代”，历史性融入珠三角2小时经济圈。“四城同创”全面推进，成功申办广东省第十七届运动会，城市发展进入品质提升新阶段。

　　改革创新深入推进。高新区晋升国家高新区，担当创新发展新使命。“两本四专”全部招生开学，加快推动人力资源转化为人才红利。“数字政府”建设全面启动，市级“最多跑一次”能办成的事项1173项，占比65.4%。上线“粤省事”微信小程序茂名专版，足不出户可办理200项民生事项，走在全省前列。

　　生态环境持续向好。市区空气质量稳居全省前三。露天矿生态公园获批国家矿山公园，生态修复经验在《人民日报》整版报道，《茂名市露天矿生态公园保护管理条例》颁布实施。茂名循环经济示范中心动工建设，绿色发展增添新动力。

　　人民生活继续改善。全市民生支出356亿元，增长10.8%，占财政支出的84.1%。城乡居民人均可支配收入增长7.4%。7578户贫困户喜迁新居，392户因灾全倒户将在春节前全部搬入新居。

　　一年来，我们主要做了以下工作。

　　（一）坚持做强实体经济，千方百计提效益、促转型

　　制造业加快转型发展。坚持抓经济必须抓项目、抓项目必须抓招商，相继举办5场高规格投资推介会，成功举行首届茂商大会，与保利集团、国药集团、中国能源等开展务实合作。实施迎接建市60周年产业行动计划，新增投资5000万元以上项目141个，新投产企业40家，天源石化丙烯项目、茂名石化150万吨/年连续重整等项目建成投产。高新区成为全国第二大环氧乙烷深加工基地，氢燃料电池、众和对位酯、新华粤金刚烷等一批新能源、新材料项目加快建设。大力发展民营经济，新增“小升规”企业80家，民营经济增加值占GDP比重达61%。成功引入中国建材、广州酒家等战略投资者，带动一批传统企业转型发展。电白建筑集团上榜“广东省百强民营企业”。全面落实“实体经济十条”等政策，建立重点工业项目服务官制度，减轻企业负担14.26亿元。在2017年度全省制造业发展分类评估中，荣获优秀等次。

　　创新驱动实现新突破。坚持创新第一动力，全社会R&D投入18.79亿元。成功申报国家农业科技园，新建4个院士工作站、3个联合实验室。申报省级工程中心21家，居粤东西北前列。新增国家高新技术企业38家。新增工业类省名牌产品7个，为历年最多。广东众和公司获颁省政府质量奖，实现该奖项在粤西地区零的突破。高分通过国家知识产权试点城市验收，获国家及广东省专利优秀奖各1项。获评3家省级劳模创新工作室。出台支持科技创新政策及“人才政策20条”，高层次人才公寓开工建设。

　　现代服务业提速发展。金融业加快发展，中信银行茂名分行开业运营。全市金融机构存贷比48.67%，提高1.92个百分点。化州金融惠民“村村通”荣获全国民生示范工程奖。旅游业提质升级，新增2家4A级景区，旅游接待突破4000万人次，旅游总收入426亿元，增长29.7%。信息服务业发展势头好，跻身全国“互联网+”城市百强榜。茂名首家跨境电商清关监管中心在电白建成运营。高州获批广东省电子商务进农村综合示范县。

　　农业加快提质增效。现代农业捷报频传，农产品加工总产值突破1000亿元，成功申报国家现代农业产业园、2个国家田园综合体试点和4个省级现代农业产业园。信宜纳入首批国家农村产业融合发展示范园。新增农业类省名牌产品19个，居全省前列。新型农业经营主体突破1.6万个，高州纳入国家农民专业合作社质量提升整县推进试点县。完成农村土地确权颁证工作。连续两年获省耕地保护考核二等奖、节约集约利用评价三等奖。

　　（二）坚持提升城市品质，不断增强城市吸引力、承载力

　　人居环境更加优化。掀起创文创卫热潮，重点实施“三整治二提升”行动，强化爱国卫生运动，城市人居环境和市民文明素养有效提升。新建和提升改造城市公厕29个。完成农贸市场改造18家。城市建成区黑臭水体消除比例80%，完成年度任务。市区垃圾无害化处理率100％。《茂名市城市市容和环境卫生管理条例》获省人大常委会批准。城市更加文明有序，得到人民群众普遍赞誉。

　　城市功能加快完善。实施迎接建市60周年城建行动计划，启动143个城建项目，完成19条城市道路升级改造和建设工作，12个道路桥梁项目加快建设。官渡公园、潘茂名纪念公园建成开放。完成一批雨污管道、雨水口改造工程，清疏城区雨污管道913.2公里。顺利通过国家园林城市省级普查，城市建成区绿地率达40.65%。成功举办首届迎春花灯会。电白新打造滨海夜景风光带。数字化城市管理平台建成运行。《茂名城市总体规划（2011-2035）》获省批复，建设宜居宜业宜游的滨海绿城有了明确指引。

　　城市经济日趋活跃。引入城市开发战略投资者，加快站南片区CBD规划建设。城光世纪城、保利中环广场、电白万达广场等城市综合体加快建设。小东江酒吧街等特色街区建设顺利推进，1959南越文创街投入使用，城市中高端消费加快培育。

　　（三）坚持厚植发展基础，全力以赴补短板、强弱项

　　交通建设稳步推进。完成交通建设投资71.6亿元，东承西接的立体交通体系加快完善。云茂高速茂名段全面建设，沈海高速茂名段扩建工程动工建设。茂名港疏港铁路即将开工。广湛客专项目可研报告通过评审。茂名综合客运枢纽南站房、南广场开工建设。茂名-高州-信宜城际列车开通运营。南排大道、中德大道等项目建成通车，茂化快线东延线加快建设，“六纵三横”快速路网基本成型。农村公路安全生命防护工程完成整治1135公里。

　　环保设施建设成效显著。4个县级绿能环保发电项目全部启动，推进力度居全省前列。全域采用PPP模式整县推进镇村污水处理设施建设。加快补齐“十二五”规划污水管网缺口，新建成各类污水管网190公里，城镇污水处理考核居全省前列。

　　水利电力短板加快补齐。中小河流治理（二期）项目全部开工，治理长度居全省首位。鉴江高岭拦河闸重建工程基本建成。村村通自来水工程、滨海新区供水工程等稳步推进。投资8.5亿元完成新一轮农网升级改造。110千伏石化南变电站建成投产，解决了长期困扰高新区发展的单回路电源问题。

　　（四）坚持改革开放引领，培育发展新优势、新动力

　　重点领域改革不断突破。启动实施10大领域54项改革任务。“放管服”改革纵深推进，“最多跑一次”“只进一扇门”审批服务日趋常态化，市行政服务中心事项进驻率83.3%，在线申办事项率87.3%。取消各类证明事项80项。企业开办时间缩短至5个工作日，全年新增市场主体4.4万户，增长26.9%。不动产登记实现一般登记5天、抵押登记3天办结，群众反映强烈的耗时长、办理难问题有效解决。高州“大病不出县”模式成为全省医改样本。启动农村“三变”改革试点。市县机构改革顺利推进。

　　开放发展取得新成绩。出台进一步扩大对外开放积极利用外资若干措施，与“一带一路”沿线新加坡、德国、阿联酋等国一批知名企业开展合作交流。与黑龙江伊春市加强干部交流和产业对接。获得2020年北部湾经合组织第11次成员大会承办权。持续提升“单一窗口”国家标准版应用覆盖率和普及率，全面提升跨境贸易便利化水平。水东港口岸更名为茂名港口岸取得突破。

　　（五）坚持推进协调发展，持续增强发展平衡性、协调性

　　三大平台再担新使命。聚焦打造沿海经济带新增长极，三大平台担当发展新使命。滨海新区聚力建设深水大港，防波堤工程顺利完工，粤电煤炭码头、通用码头、博贺湾大桥、10万吨级航道等项目加快建设。粤电博贺电厂保护性复工，益海嘉里项目动工建设，临港经济蓄势崛起。博贺新港区加快开港前的准备工作，现代化深水大港呼之欲出。高新区聚力发展高新产业，中准新材料等7个项目建成投产，绿色智慧大数据等14个项目动工建设，代表未来方向的新产业加快突破。共青河新城设计及控规成果通过专家评审。水东湾新城聚力发展滨海旅游，水东湾大桥加快建设，水东湾海洋公园及歌美海公园一期工程完成建设，水质净化厂等21个项目破土动工，正成为广东滨海旅游“新明珠”。

　　县域经济稳步发展。工业园区扩能增效，新增2个产业集聚地。6个省级园区投产项目24个，承接珠三角梯度转移项目30个，实现规上工业增加值114亿元，增长10.9%。县域副中心加快建设，“十件实事”全面铺开。乡村振兴开局良好，农村人居环境综合整治扎实推进，省定贫困村创建新农村示范村启动建设项目1.01万个。特色小镇建设实现新突破，化州新安镇成为全省唯一的国家小城镇建设试点，电白沙琅镇入选全省“十大明星小镇”。

　　（六）坚持绿色发展，坚决守护好“茂名蓝”、生态美

　　环保督察整改顺利推进。中央环保督察“回头看”交办我市案件全部办结，省环保督察交办案件办结率达95.7%。积极配合国家海洋督察整改。基本完成茂名石化炼油厂卫生防护距离一期二批搬迁安置工程，推进成效得到中央第五环保督察组充分肯定。

　　重点领域污染防治成效明显。切实担负起环境保护政治责任，全面推进污染源普查工作，制定大气、水、土壤、固体废物等领域污染防治攻坚方案。整治“散乱污”企业110家。清理整治养殖场5808家，《茂名市畜禽养殖污染防治条例》获省人大常委会审查批准。严厉打击非法处置固废危废行为，责令整改53宗、立案49宗。城市集中式饮用水水源水质达标率保持100%。地表水断面水质优良率达85.7%。入海河流水质稳中向好。

　　环保工作机制加快完善。建立湖长制、湾长制，河长制工作成效得到国家评估组高度肯定。组建治安支队食药环大队，强化环保和公安联动执法。制定办理环境公益诉讼案件衔接实施办法，依法打击环境违法行为。创建国家森林城市顺利推进，在2017年度全省森林资源保护和发展目标责任制考核中荣获第二名。

　　（七）坚持增进人民福祉，不断提升群众获得感、幸福感

　　社会保障水平再提升。全市新增城镇就业6.5万人。“粤菜师傅”工程全面启动，遴选建设16家培训基地。城乡居民医保大病保险最高支付限额提高到30万元，特困供养人员大病保险支付比例由80%提高到90%。企业职工养老保险基金实现省级统筹，有力保障我市企业退休人员养老待遇足额发放。特困人员供养、城乡低保、孤儿基本生活保障、残疾人“两项补贴”发放等顺利开展。坚决打好精准脱贫攻坚战，三年投入财政专项扶贫资金16.97亿元，6.73万户15.49万人达到2018年预脱贫标准。超额完成棚户区改造任务。成功应对“8·11”暴雨洪涝和“山竹”强台风灾害。

　　教育卫生事业提质发展。支持教育优先发展，全年教育投入123.88亿元，增长6.6%。成为全省“创建国家级农村职业教育和成人教育示范县”最多的地市。教育创现进入省督导验收阶段。技工教育成效明显，首次获得技能竞赛国际大奖。全面铺开13家县级公立医院升级建设，完成64家乡镇卫生院标准化建设，基层医疗卫生服务能力再提质。新增社会办医疗机构57间、床位358张。

　　文化事业繁荣发展。成功举办第三届“朝圣敦煌”全国美术作品展（茂名站）、中国书协理事作品展等“好心茂名”系列展览，以及首届冼夫人文化周、国际生态马拉松赛、“好心茂名”龙舟邀请赛等文体活动，评选茂名十大文化名片，打响“好心茂名”城市品牌。出台《茂名市冼夫人文化发展纲要》，拍摄纪录片《海丝·茂名》。全市村（居）综合性文化服务中心建设完成率居粤东西北首位。

　　社会治理稳步向好。以扫黑除恶专项斗争为契机，深入开展“飓风2018”、护航金融“利剑行动”、禁毒“粤剑扫毒”等专项行动，全面加强基层派出所建设，全市“两抢一盗”发案率下降21.12%。电白成功摘掉“毒品滥用危害严重地区”帽子。深化危化品、道路交通、公安消防等重点领域安全治理，安全生产形势保持稳定。高州获评全国法治县（市）创建活动先进单位。突发事件应对能力有效加强。全链条食品药品监管体系进一步筑牢。工会、共青团、妇联等群团改革稳步开展，桥梁纽带作用充分发挥。同时，外事、侨务、民族、宗教、人防、打假、打私、地震、气象、经济普查、国防动员、双拥优抚等工作都取得新成绩。

　　注重政府自身建设。把政治建设摆在首位，坚持以习近平新时代中国特色社会主义思想引领政治站位、引领价值取向、引领意志品质。强化法治政府建设，认真开展政府立法工作，提出2个法规议案，制定20件规范性文件。坚持依法行政，认真执行政府常务会学法计划，全年办理行政复议案件117件，纠正一批违法行政行为，获省依法行政考评良好等次。主动接受人大依法监督和政协民主监督，办理人大建议129件、政协提案201件，办复率100%。驰而不息落实中央八项规定精神。政务公开更加透明、更加深化。强化审计监督、舆论监督，持续打造风清气正的政务环境。

　　各位代表！过去一年的成绩来之不易！这是省委、省政府和市委正确领导、科学决策的结果，是市人大、市政协和社会各界守望相助、鼎力支持的结果，是全市人民同心同德、奋力拼搏的结果。在此，我代表市人民政府，向辛勤工作在各行各业的广大干部群众，向大力支持政府工作的人大代表、政协委员、各民主党派、无党派人士、各人民团体和社会各界人士，向驻茂人民解放军、武警部队官兵、驻茂单位，向积极参与茂名建设的港澳台同胞、海外侨胞及国际友人，表示衷心的感谢和崇高的敬意！

　　我们也清醒地认识到，工作中还存在一些不容忽视的问题：一是经济下行压力加大，受宏观经济形势及我市工业投资放缓等影响，规上工业增加值、固定资产投资等个别指标未达到年初目标；二是财政收支矛盾较突出，重点项目、民生保障方面刚性支出不断增加，收支平衡难度大；三是发展基础薄弱，土地、资金、人才等要素制约突出；四是工业发展后劲不足；五是民生保障水平与人民群众期待仍有较大差距；六是政府自身建设需要进一步加强，少数干部不作为不担当现象仍然存在，营商环境有待改善。对此，我们将切实负起责任，采取更加有力的措施加以解决。

　　二、2019年工作安排

　　各位代表！今年是新中国成立70周年，是全面建成小康社会的关键之年，也是茂名建市60周年。站在新的历史起点上，纵观当前发展形势，国际环境深刻变化，我国经济从高速增长转向高质量发展的态势更加明显，经济运行稳中有变、变中有忧，发展不平衡不充分问题和各种周期性、结构性问题交织叠加；地区间竞争激烈，分化趋势更加突出，营商环境从“硬实力”的较量转向“软实力”的比拼；茂名正处在爬坡过坎的关键时期，经济下行压力较大，补短板、固底板、强弱项的紧迫感与日俱增。

　　面临的困难与挑战不可低估，但更要看到机遇和希望。只要我们保持战略定力，集中精力办好自己的事情，我们有信心、有能力克服困难、战胜挑战。我们的信心和力量，来自习近平新时代中国特色社会主义思想的强大真理力量、实践力量；来自党中央作出我国发展仍处于并将长期处于重要战略机遇期的科学判断；来自省委省政府构建“一核一带一区”区域发展格局，把茂名作为沿海经济带新增长极的历史机遇；来自富集的石化、海洋、农业、生态等资源以及由此带来的巨大发展潜力；来自交通、城市发展瓶颈逐步突破后，越来越多的市场主体关注茂名、看好茂名、投资茂名；来自好心茂名城市文化凝聚的强大精神力量。“信心比黄金更重要”，只要我们坚定信心，担当作为，就没有迈不过的坎、克服不了的困难，就一定能完成既定各项目标任务。做好今年政府工作，要把握好以下几点：一是坚持稳中求进工作总基调。主动适应和把握稳中有变新形势，着力推进“六个稳”，确保发展大局稳。在“稳”的基础上抓改革、破难题、增活力，推动新技术新产业发展应用，激发“进”的动力。二是坚持抓经济必须抓产业。树立大抓产业鲜明导向，聚焦招大引强，开展全行业全领域招商引资，筑牢茂名高质量发展产业根基。三是坚持向改革创新要动力。深化经济功能区、科技创新、投融资体制机制改革，建立健全抓落实长效机制，以优质的制度供给、服务供给，增强城市吸引力和竞争力。四是坚持打好三大攻坚战。坚决防范重大风险，把提高脱贫质量放在精准脱贫工作首位，推动生态环境持续好转，夯实高质量发展基础。五是坚持融入国家和省重大战略。把粤港澳大湾区建设作为茂名改革开放的大机遇，精准对接大湾区产业转移、资源溢出，打造大湾区向西拓展的重要腹地。与北部湾经济区、海南自贸区相向而行，借力借势谋发展。六是坚持以人民为中心。把就业、教育、医疗、社保、住房等问题一个一个解决好，更好满足人民日益增长的美好生活需要，让人民群众获得感、幸福感、安全感更加充实、更有保障、更可持续。

　　做好今年政府工作，要坚持以习近平新时代中国特色社会主义思想为指导，全面贯彻党的十九大和中央经济工作会议精神，深入贯彻习近平总书记对广东重要讲话和重要指示批示精神以及省委十二届六次全会精神和李希书记调研茂名讲话精神，按照市委十一届八次全会“1+4+6”工作布局，牢牢把握稳中求进工作总基调，坚持新发展理念，坚持推动高质量发展，以供给侧结构性改革为主线，围绕“巩固、增强、提升、畅通”八字方针，大抓产业建设，深化改革开放，提升城市品质，加快乡村振兴，践行绿色发展，增进民生福祉，建设好心茂名，攻坚全面建成小康社会，奋力推动茂名加快形成广东沿海经济带上的新增长极。

　　今年经济社会发展主要预期目标是：地区生产总值增长6.5%-7.0%，固定资产投资增长7%，社会消费品零售总额增长8.5%，一般公共预算收入增长6%，居民收入增长与经济增长同步，单位生产总值能耗、主要污染物总量完成省下达的目标任务。

　　今年要重点抓好以下九方面工作：

　　（一）持之以恒做强实体经济，夯实高质量发展的产业根基

　　大抓产业项目建设。旗帜鲜明抓产业兴产业，加快经济发展质量变革、效率变革、动力变革。全力推进纳入2019年重点项目库的67项产业项目建设，力争完成年度投资149.5亿元。建立要素保障机制，力保土地、资金、政策向各类产业项目倾斜。探索设立产业投资基金，支持符合规划的产业项目建设。鼓励众和、新华粤、茂化实华等本土优势企业延链补链，上马化工新材料、精细化工项目。推动茂名石化炼油产品结构优化项目等大项目建成投产，力争有更多新的重大产业项目落地。抢抓港口、机场、高铁发力建设的机遇，大力发展现代服务业，建设高水平粤西空港经济区、博贺新港区综合物流园。加快发展工业互联网，与大数据、电子商务、智能制造等有机结合、互促共进。

　　全力抓好招商引资。把招商引资作为“一号工程”，坚持引入有理念、有经验、有实力的大型知名企业。加强招商引资领导力量和组织力量，建立完善招商引资联席会议制度、考核奖励制度、项目跟踪服务制度。办好临港产业、城市经济等系列精准招商活动，找准符合产业规划的市场主体。围绕医药产业优势，积极推动8大医药与健康产业项目落地。充分利用国家和省的各项产业政策，坚持“一事一议”“一企一策”，推动更多意向项目转为落地项目。借助各地商会、行业协会、贸促会等专业组织的力量，拓宽招商渠道，提高招商实效。全力支持乡贤回归创业。

　　聚焦园区提质增效。把今年定为“园区提质建设年”，集中攻坚园区发展瓶颈。探索引入市场化力量，全面加快基础设施建设，提高园区承载力。通过“放管服”改革赋予园区部分县级审批职能，夯实园区公共服务平台。大力推进园区批而未供、供而未用土地处置，提高土地节约集约利用水平。打破零敲碎打承接低端产业转移的传统模式，走好产业集群发展之路，今年力争各省级园区均引入3-5个投资超亿元产业项目，全市省级园区规上工业增加值增长10%以上。因地制宜建设特色产业园，茂南重点建设高岭土产业园、石化产业园，电白重点建设绿色建筑产业园、香精香料产业园，信宜重点建设水口高新科技园，高州重点建设医药健康产业园，化州重点建设杨梅官桥产业园。

　　提升科技创新水平。聚焦提高全社会研发投入，加快氢能源高新技术研发中心等创新平台建设，推动院士工作站、联合实验室早出成果，争取省重大创新平台落户茂名。强化孵化育成体系建设，推动大众创业万众创新。支持广东石油化工学院建设高水平理工科大学。创新柔性引才机制，与著名高校和科研院所开展多形式科研合作。强化知识产权保护，争创国家知识产权示范培育城市。开展质量提升行动，推动制造业提品质、创名牌、增品种，创建质量强市示范城市。出台急需紧缺人才管理办法。完善高层次人才数据库。加快人才公寓建设。

　　大力支持民营经济。成立以市主要领导为组长的促进民营经济发展领导小组，致力解决“四难”问题。对融资难问题，组建中小微企业转贷基金，做大中小微企业贷款风险补偿“资金池”。引入1家专业融资担保机构，做大政策性融资担保业务规模。对暂时遇到困难的企业千方百计给予支持，防止随意抽贷、断贷、停贷。对用地难问题，支持本土企业增资扩产，在项目审批、要素保障方面开通绿色通道，研究出台解决中小企业用地难的政策措施。对用工难问题，扶持引导高校毕业生、就业困难人员、农村转移劳动力等重点群体就业创业。拓宽校企合作渠道。研究出台留住“两本四专”高校毕业生在茂就业政策举措。对降成本难问题，抓好省新“实体经济十条”“民营经济十条”等政策落地，出台我市实施细则。全面贯彻减税降费政策，清理规范中介机构收费行为。坚决落实重点工业项目服务官制度，抓好“个转企、小升规”工作。强化政府履约意识，依法审慎采取强制措施，保护企业家合法权益和人身财产安全。构建“亲”清”新型政商关系，做到亲而有度、清而有为，帮忙不添乱、履职要尽责。

　　（二）坚定不移发展向海经济，打造沿海经济带新增长极

　　强化交通先导地位。引入高水平团队编制全市综合交通运输规划。完成茂名综合客运枢纽一期工程，启动二期工程建设。全面建设茂名港疏港铁路，全力配合广湛客专建设。加快建设云茂高速茂名段，抓好沈海高速茂名段扩建工程，推进中山至高州（粤桂界）高速公路前期工作。加快实施列入省规划的普通国省道升级改造工程，争取年内全部开工。推进博贺湾大桥、水东湾大桥建设，加快茂化快线东延线、复兴大道等交通项目建设。

　　加快做强深水大港。把港口作为打造向海经济的战略抓手，全速推进博贺新港建设，优化水东港功能。完成茂名港总体规划修编报批工作。建成10万吨级航道、粤电煤炭码头、通用码头和港政码头，抓紧建设口岸通关服务中心、供水供电工程，确保博贺新港区今年3月如期开港。加快壮大临港工业，推进博贺电厂及配套电网工程建设，加快益海嘉里项目建设。启动吉达港区开发建设，力促丙烷脱氢项目落地。争取开工建设博贺新港东区油品及化工码头、昌利油品码头、利丰码头项目。抓紧30万吨级原油码头、30万吨级航道等项目前期工作。加快LNG造陆工程、燃气管道、公共管廊等设施建设。进一步完善博贺湾新城功能配套。

　　高水平建设高新区。推动高新区争先晋位，担当引领茂名高质量发展的责任和使命。实施好深化高新区体制机制改革的意见，强化引入高层次专业人才和研发队伍的政策措施。抓紧推进氢燃料电池、环保印刷油墨等项目建设，争取引入新能源汽车、中华煤气生物能源等新产业项目。加快新材料、新能源、大数据和生物健康等新兴产业发展。大力发展环氧乙烷、医药中间体、日化材料等产业链项目。做强科技创新平台，引导创新要素加快集聚。

　　聚力发展滨海旅游。立足优质滨海资源，全力打造南海博贺国家级滨海旅游度假区。开工建设广东滨海旅游公路茂名段。全力抓好南海旅游岛土地盘整收储，强化滨海旅游规划管控。加快华侨城等项目建设，争取新加坡鹏瑞利康养综合体项目、海昌海洋公园项目落地建设，推动单一景区向旅游康养一体化发展。加快创建电白、信宜全域旅游示范区。推进水东湾蓝色海湾建设。强化智慧旅游建设，完善旅游厕所等旅游基础设施配套。推动山海旅游融合发展，打响“山海之旅、心灵之约”旅游名片。

　　（三）纵深推进改革开放，增强内生发展的动力活力

　　深化“放管服”改革。全面加快“数字政府”建设，创建政务服务“三大品牌”、建设“三大服务平台”、做好“四大主题服务”，扎实推进审批服务“马上办、网上办、就近办、一次办”，努力解决办事难、办事繁问题。加快工程项目审批制度改革，推行并联审批、联合评审和投资项目承诺制，确保工程项目总体审批时间压减到45个工作日。提升企业开办便利度，全面推进企业简易注销登记改革。推动“双随机、一公开”监管常态化。持续精简行政许可和各类证明事项，成熟一批、推出一批。

　　推进财政、金融和投融资体制改革。做好省以下财政体制改革，实施预算管理改革。规范政府举债融资机制，积极争取地方政府新增债券额度，防范重大债务风险。完成农村信用社改革，力促交通银行茂名分行建成开业，争取今年全市金融机构存贷比超50%。支持企业拓展资本市场融资渠道。加快金融监管体制改革，严厉打击乱办金融、非法集资活动，坚决守住不发生系统性金融风险的底线。抓住国家促进基建的有利时机，健全市场化投融资体制机制。推进招标投标改革。推动城投集团、交投集团、茂名港集团等企业在市场竞争中发展壮大，确保国有资本保值增值。

　　蹄疾步稳推进机构改革。完成市县机构改革，出台“三定”方案，科学配置行政资源，推进市县治理体系和治理能力现代化。全面推进承担行政职能的事业单位改革。继续完善经济功能区和县域副中心体制机制。

　　构建开放型经济新格局。以沿海经济带建设为抓手，积极对接融入粤港澳大湾区，拓展茂名能有所作为的政策空间。与北部湾经济区、海南自贸区相向而行，联手打造中国滨海旅游目的地和现代农业发展集聚区。密切与黑龙江省伊春市对口合作关系。加强与新加坡、德国等“一带一路”沿线国家经贸往来。

　　（四）聚力建设品质城市，提高城市首位度和承载力

　　推进品质城市建设。重新审视城市发展的历史文脉、规律特点，提升城市建设现代感和文化传承性。强化城市设计理念，健全城市控制性详细规划体系，增强城市建筑立体感、色彩感。加快站南片区开发建设。推进城光世纪城、五洲广场等城市综合体建设。启动茂名影剧院、锦堂村等“三旧”改造工程。规划建设市文化中心、美术馆、大剧院等高品质文化基础设施。完善西城片区规划设计。推进电白水东新片区“三公园一广场”建设。

　　着力完善城市功能。加快创文创卫惠民利民，推动形成人人出力、共筑和谐美丽家园的氛围，高水平完成年度创建任务。攻坚迎接建市60周年城建项目，实施“八公园、八小区、十二道路”建设工程。新建和改造升级城市公厕40个。完成14家农贸市场升级改造。继续完善城市慢行系统。加快老旧小区、背街小巷微改造。抓好城区临时停车泊位建设。推进红旗北路夜市等特色街区建设，促进城市消费。完成地下综合管廊、城区排水及防洪、排涝等专项规划编制工作。稳步建设海绵城市，增强城市韧性。

　　加强城市精细化管理。用好数字城管平台，推动城市高效运行，以绣花功夫抓好城市细节管理，整治城市管理黑点，“绣”出城市新面貌。继续推进垃圾分类收集处理。加强社区治理、小区物业和农贸市场管理。推动《茂名市物业管理办法》立法。加快市政设施市场化、园林养护社会化。深入推进爱国卫生运动。

　　启动建设共青河新城。抓紧出台共青河新城城市设计及控规方案。谋划中央公园奥林匹克湖建设，启动奥体大道等5条道路建设，加快奥体中心等7个项目建设。

　　（五）统筹城乡融合发展，打造全省乡村振兴的“茂名样板”

　　扎实建设现代农业产业园。现代农业产业园是新时代“三高”农业升级版，要以现代农业产业园、田园综合体试点为龙头，创建一二三产业融合发展先导区，让农业成为有奔头的产业。建立完善产业园规划、设计、建设和管理运营一体化机制，形成政府引导、市场主体、多方投入发展格局。整合涉农资金，向建设快、成效好的园区倾斜。加快培育一批懂农业、爱农村的新型农民。

　　加快完善农村基础设施。以“千万工程”为引领，扎实推进农村人居环境综合整治，打造一批宜居美丽乡村、渔村。全面加快“四好农村路”建设，筑牢农村公路安全生命防护工程。抓好农村“四边”垃圾治理。开展“平安村口”建设。继续推进中小河流治理工程，优化区域水资源配置。加大电网建设投入，推进农村电网升级改造，改善信宜东部五镇中压网架。扎实推进拆旧复垦和垦造水田工作。继续做实做强4个县域副中心。

　　打好精准脱贫这场硬仗。坚持市县镇村四级书记抓扶贫，一鼓作气、尽锐出战，深入推进180条省定贫困村创建新农村示范村。推进教育医疗、危房改造、社会救助扶贫，实现应扶尽扶。把防止返贫摆在更加重要的位置，大力支持扶贫产业基地建设，突出解决“两不愁三保障”问题，做好兜底工作。抓好与南宁、来宾“携手奔小康”扶贫协作。强化考核监督，抓好扶贫资金使用和项目建设，关心爱护基层扶贫干部，坚决打赢脱贫攻坚战。

　　做强做优特色农业。以“产业兴旺”为乡村振兴之本，推动特色农业高质量发展。突出抓好农民合作社和家庭农场。建设10个50亩以上农业科技示范基地。大力发展“一村一品、一镇一业”，对荔枝、化橘红、沉香等拳头产品进行品种改良和品牌塑造，打响“茂字号”品牌。大力实施渔港振兴计划。落实粮食安全责任制。抓好农村土地和集体产权制度改革，加快农村“三变”改革。提升动物防疫能力建设，严防死守抓好非洲猪瘟防控工作。

　　（六）致力建设美丽茂名，让碧水蓝天成为最鲜明的城市特色

　　锲而不舍抓紧督察整改。以督察整改倒逼生态环境改善，扎实推进城市黑臭水体、固废危废监管、镇级垃圾填埋场等突出问题整治。全面完成被督察通报的污水管网建设及饮用水源保护区问题整治任务。抓好交办案件办理工作，严防敷衍整改、虚假整改。

　　加快污染防治产业化。研究制定绿色低碳产业发展规划。全面建设茂名循环经济示范中心项目。加快建设4个绿能环保发电项目。全面推进PPP模式整县推进镇村污水处理设施建设。引入新技术推进餐厨垃圾处理、畜禽养殖废弃物资源化利用，争取规模养殖场粪污处理设施装备配套率达80%以上。严格执行企业排放标准，加快节能环保技术推广应用。

　　全力解决环境突出问题。突出抓好水污染防治，坚决落实省“一号令”，消除劣Ⅴ类水质断面。持续加大投入、严格管理，保护好高州水库饮用水源。严厉打击非法倾倒转移固废危废行为。完成VOCs企业落后产能淘汰退出、生物质成型燃料锅炉和“散乱污”企业专项整治。完成全国第二次污染源普查，开展土壤污染状况详查。大力推进茂名石化炼油厂卫生防护距离内居民搬迁安置工作。攻坚国家森林城市创建，完成3.7万亩森林碳汇工程、132个乡村绿化美化示范点建设。积极开展“万里碧道”试点工程。

　　同时，强化领导干部任期生态文明建设责任制。健全生态保护补偿机制，完善财税激励政策。加强生态环境队伍建设，提升执法能力，打造一支政治强、作风硬、敢担当的生态环境保护铁军。

　　（七）用心建设“好心茂名”，以文化的力量影响人激励人

　　广泛普及好心文化。大力弘扬“好心茂名”城市精神，全市各行各业要争做好心人、办好心事，做传播好心文化的使者。深入挖掘冼夫人、潘茂名文化资源。深入开展道德模范、茂名好人评选活动。支持建设“茂名好人馆”，加快建设茂名英烈纪念馆。推进新时代文明实践中心建设。围绕新中国成立70周年、茂名建市60周年开展好心文化创作展演，营造向上向善的文化氛围。

　　大力繁荣文体事业。高水平办好“好心茂名”龙舟邀请赛、茂名国际生态马拉松、广东龙舟锦标赛、第二届北部湾城市运动会等大型赛事。推进市级全媒体中心和高州、信宜县级融媒体中心建设。加强体育人才培养，积极筹备省运会。办好建市60周年系列文体活动，聚人心、展形象，凝聚推动茂名振兴发展的澎湃力量。

　　深入推进文旅融合。找准文化旅游融合点，挖掘冼夫人、年例、地方非物质文化遗产等文化资源，打造富有地域特色的文旅产品。推动冼太故里景区创建全国研学旅游示范基地。支持高州冼太庙申报全国文物保护单位。积极推进《冼夫人文化发展纲要》确立的“十个一工程”建设，擦亮冼夫人文化名片。办好南海（茂名）博贺开渔节、荔枝文化节等节庆活动。

　　（八）持续增进民生福祉，努力让人民群众共享发展成果

　　织密扎牢社会保障网。民生是为政之要，切实办好“省十件民生实事”。抓好就业这个最大民生，加强公共就业平台建设，落实积极就业政策。大力开展“粤菜师傅”工程。继续推进社保扩面征缴工作。探索推进医保城乡一体化改革。完善医疗救助制度。按“五有”要求完善退役军人服务管理体系，维护退役军人合法权益。扶持养老事业发展，完善颐年苑配套建设，引入社会资本参与敬老院运营管理。推进保障性住房建设，加快共有产权住房试点工作，健全城镇住房保障体系。全力完成农村危房改造任务。

　　尽心尽力发展教育卫生事业。保障教育优先发展，落实中小学教师福利待遇。加快市直学校10个改扩建项目建设，建成崇文学堂、东江学堂，新增7000个优质学位。高度重视学前教育，开工建设市第三幼儿园，严格落实住宅小区配建幼儿园规定。深化“县管校聘”改革。完成“广东省推进教育现代化先进市”申报验收工作。继续抓好“两本四专”提质建设和内涵式发展，推进茂名技师学院申报高等职业院校。落实健康中国战略，扎实推进基本公共卫生服务，抓好重大传染病防控工作。完成3个县级公立医院升级改造。建成市中心医院一期工程，加快中医院新院区建设。加强妇幼保健能力建设。支持社会资本参与医疗事业发展。

　　大力创建平安茂名。坚决打好扫黑除恶专项斗争，持续严厉打击涉毒、涉枪、涉电信诈骗等突出违法犯罪活动，强化公安基层基础建设，促进社会治安形势持续向好。推进智慧警务建设。践行新时代“枫桥经验”，建立健全基层社会矛盾预防和化解体制机制，强化农村基层社会治理。依法加强网络空间治理。出台党政领导干部安全生产责任制细则，全面压实领导责任，坚决压实企业生产安全主体责任。铁腕整治危化品、道路交通、消防等领域安全隐患，坚决遏制较大以上生产安全事故。加强应急能力建设，加快建设一支专业化应急救援队伍。建成茂名石化产品运输车辆综合服务中心。强化食品药品安全监管。高质量完成第四次经济普查工作。抓好工青妇、民族宗教、国防动员、双拥优抚、外事、侨务、人防、气象、地震、残疾人、红十字等工作。

　　（九）加强政府自身建设，努力建设为民务实清廉的人民政府

　　旗帜鲜明讲政治。坚决在思想上政治上行动上与以习近平同志为核心的党中央保持高度一致，树牢“四个意识”，坚定“四个自信”，落实“两个维护”。始终牢记党性原则，扎实开展“不忘初心，牢记使命”主题教育，认真落实“三会一课”、民主生活会、谈心谈话等制度，持续净化党内风气，以政治建设的坚定有力，推动中央、省、市决策部署不折不扣得到落实。

　　担当作为促发展。面对茂名打造沿海经济带新增长极的紧迫任务和难得机遇，我们要始终怀抱强烈的事业心、坚定的使命感，义不容辞扛起振兴茂名的责任。要敢啃产业发展的硬骨头，滴水穿石，久久为功，积小成为大成。要敢破项目建设的艰难险阻，盯住不放，雷厉风行，不见成效不收兵。要以事业为导向，用实绩论英雄，把干部干了什么事、干了多少事、干的事群众认不认可作为选拔任用干部的根本标准，让敢担当善作为的干部有舞台、受褒奖，让不作为、慢作为的干部让位子、摘帽子，形成齐心干事、合力成事的工作氛围。要落实“三个区分开来”要求，理直气壮为担当作为的干部撑腰鼓劲，让他们干得有热情、有底气、有奔头。我们要增强“八大本领”，弥补知识空白、经验盲区、能力弱项，锻造担当作为的“茂名力量”。

　　依法行政作表率。以建设法治政府为目标，坚持依法行政、依法执政，政府系统各单位主要负责人要切实履行法治政府建设第一责任人职责。充分发挥政府法律顾问室和法律顾问在推进依法行政中的积极作用。自觉接受人大依法监督、政协民主监督和审计监督、舆论监督，办好人大代表建议和政协委员提案。强化政务公开，推进政策解读和网络问政工作。

　　廉洁奉公守底线。坚决把管党治党的政治责任作为根本政治担当，坚持严字当头、全面从严、一严到底，锲而不舍落实中央八项规定及其实施细则精神。坚决把心思放在工作上，把精力放在发展上，把时间放在落实上，反对表态多调门高、行动少落实差等形式主义、官僚主义。我们要知敬畏、存戒惧、守底线，习惯在制度约束和监督的环境中工作生活。

　　各位代表！2019年有机遇也有挑战，大家还要一起拼搏、一起奋斗。让我们紧密团结在以习近平同志为核心的党中央周围，高举习近平新时代中国特色社会主义思想伟大旗帜，闻鸡起舞、日夜兼程、风雨无阻，全力以赴把茂名的事情办好，把时代交给我们的考题一个一个解答好，奋力建设产业实力雄厚的现代化滨海城市，打造沿海经济带上的新增长极，以优异成绩献礼中华人民共和国成立70周年，庆祝茂名建市60周年！
